# Supplementary material for: Pyruvate Dehydrogenase Kinase 1 inhibition mediated oxidative phosphorylation enhancement in cartilage promotes osteoarthritis progression
Source: BMC Musculoskelet Disord. 2023 Jul 20;24:597. doi: 10.1186/s12891-023-06585-6 (PMC10357736; doi:10.1186/s12891-023-06585-6)
Supplement: Supplementary file 1 — Additional file 1. [file 12891_2023_6585_MOESM1_ESM.zip › BP.Gsea.1653623667859/GOBP_REGULATION_OF_RNA_SPLICING.html]

Details for gene set GOBP\_REGULATION\_OF\_RNA\_SPLICING[GSEA]

|  || Dataset | 55457\_collapsed\_to\_symbols.cls.cls #OA\_versus\_normal.cls.cls #OA\_versus\_normal\_repos |
| Phenotype | cls.cls#OA\_versus\_normal\_repos |
| Upregulated in class | normal |
| GeneSet | GOBP\_REGULATION\_OF\_RNA\_SPLICING |
| Enrichment Score (ES) | -0.5059025 |
| Normalized Enrichment Score (NES) | -2.286872 |
| Nominal p-value | 0.0 |
| FDR q-value | 0.0012143214 |
| FWER p-Value | 0.002 |
Table: GSEA Results Summary

  

Fig 1: Enrichment plot: GOBP\_REGULATION\_OF\_RNA\_SPLICING      
 Profile of the Running ES Score & Positions of GeneSet Members on the Rank Ordered List

  

| SYMBOL | TITLE | RANK IN GENE LIST | RANK METRIC SCORE | RUNNING ES | CORE ENRICHMENT || 1 | FAM172A | family with sequence similarity 172 member A [Source:HGNC Symbol;Acc:HGNC:25365] | 251 | 0.785 | -0.0036 | No |
| 2 | SRSF9 | serine and arginine rich splicing factor 9 [Source:HGNC Symbol;Acc:HGNC:10791] | 882 | 0.548 | -0.0417 | No |
| 3 | SAP18 | Sin3A associated protein 18 [Source:HGNC Symbol;Acc:HGNC:10530] | 1099 | 0.496 | -0.0484 | No |
| 4 | MBNL3 | muscleblind like splicing regulator 3 [Source:HGNC Symbol;Acc:HGNC:20564] | 1165 | 0.483 | -0.0436 | No |
| 5 | RBM23 | RNA binding motif protein 23 [Source:HGNC Symbol;Acc:HGNC:20155] | 1678 | 0.402 | -0.0754 | No |
| 6 | BRDT | bromodomain testis associated [Source:HGNC Symbol;Acc:HGNC:1105] | 1812 | 0.382 | -0.0780 | No |
| 7 | FASTK | Fas activated serine/threonine kinase [Source:HGNC Symbol;Acc:HGNC:24676] | 1834 | 0.380 | -0.0718 | No |
| 8 | RBM4 | RNA binding motif protein 4 [Source:HGNC Symbol;Acc:HGNC:9901] | 1970 | 0.364 | -0.0749 | No |
| 9 | ESRP1 | epithelial splicing regulatory protein 1 [Source:HGNC Symbol;Acc:HGNC:25966] | 2064 | 0.355 | -0.0749 | No |
| 10 | FAM50A | family with sequence similarity 50 member A [Source:HGNC Symbol;Acc:HGNC:18786] | 2203 | 0.338 | -0.0788 | No |
| 11 | ESRP2 | epithelial splicing regulatory protein 2 [Source:HGNC Symbol;Acc:HGNC:26152] | 2435 | 0.310 | -0.0905 | No |
| 12 | RBM7 | RNA binding motif protein 7 [Source:HGNC Symbol;Acc:HGNC:9904] | 2620 | 0.288 | -0.0990 | No |
| 13 | AHNAK2 | AHNAK nucleoprotein 2 [Source:HGNC Symbol;Acc:HGNC:20125] | 2738 | 0.276 | -0.1025 | No |
| 14 | RAVER2 | "ribonucleoprotein, PTB binding 2 [Source:HGNC Symbol;Acc:HGNC:25577]" | 2956 | 0.254 | -0.1143 | No |
| 15 | FXR1 | FMR1 autosomal homolog 1 [Source:HGNC Symbol;Acc:HGNC:4023] | 3012 | 0.249 | -0.1135 | No |
| 16 | C1QBP | complement C1q binding protein [Source:HGNC Symbol;Acc:HGNC:1243] | 3083 | 0.241 | -0.1141 | No |
| 17 | PUF60 | poly(U) binding splicing factor 60 [Source:HGNC Symbol;Acc:HGNC:17042] | 3237 | 0.227 | -0.1214 | No |
| 18 | SON | SON DNA and RNA binding protein [Source:HGNC Symbol;Acc:HGNC:11183] | 3374 | 0.214 | -0.1276 | No |
| 19 | LARP7 | "La ribonucleoprotein 7, transcriptional regulator [Source:HGNC Symbol;Acc:HGNC:24912]" | 3395 | 0.212 | -0.1249 | No |
| 20 | CIRBP | cold inducible RNA binding protein [Source:HGNC Symbol;Acc:HGNC:1982] | 3441 | 0.207 | -0.1241 | No |
| 21 | RBFOX2 | RNA binding fox-1 homolog 2 [Source:HGNC Symbol;Acc:HGNC:9906] | 3523 | 0.200 | -0.1264 | No |
| 22 | MBNL2 | muscleblind like splicing regulator 2 [Source:HGNC Symbol;Acc:HGNC:16746] | 3524 | 0.200 | -0.1223 | No |
| 23 | KHDRBS3 | "KH RNA binding domain containing, signal transduction associated 3 [Source:HGNC Symbol;Acc:HGNC:18117]" | 3714 | 0.184 | -0.1333 | No |
| 24 | KHDRBS1 | "KH RNA binding domain containing, signal transduction associated 1 [Source:HGNC Symbol;Acc:HGNC:18116]" | 3748 | 0.182 | -0.1322 | No |
| 25 | TMBIM6 | transmembrane BAX inhibitor motif containing 6 [Source:HGNC Symbol;Acc:HGNC:11723] | 4045 | 0.152 | -0.1522 | No |
| 26 | MAGOH | "mago homolog, exon junction complex subunit [Source:HGNC Symbol;Acc:HGNC:6815]" | 4266 | 0.131 | -0.1668 | No |
| 27 | KHDRBS2 | "KH RNA binding domain containing, signal transduction associated 2 [Source:HGNC Symbol;Acc:HGNC:18114]" | 4550 | 0.109 | -0.1867 | No |
| 28 | PCBP4 | poly(rC) binding protein 4 [Source:HGNC Symbol;Acc:HGNC:8652] | 4577 | 0.107 | -0.1865 | No |
| 29 | PTBP1 | polypyrimidine tract binding protein 1 [Source:HGNC Symbol;Acc:HGNC:9583] | 4650 | 0.100 | -0.1901 | No |
| 30 | GRSF1 | G-rich RNA sequence binding factor 1 [Source:HGNC Symbol;Acc:HGNC:4610] | 4660 | 0.099 | -0.1888 | No |
| 31 | RPS26 | ribosomal protein S26 [Source:HGNC Symbol;Acc:HGNC:10414] | 5368 | 0.043 | -0.2433 | No |
| 32 | SNW1 | SNW domain containing 1 [Source:HGNC Symbol;Acc:HGNC:16696] | 5472 | 0.035 | -0.2506 | No |
| 33 | ERN1 | endoplasmic reticulum to nucleus signaling 1 [Source:HGNC Symbol;Acc:HGNC:3449] | 5658 | 0.019 | -0.2647 | No |
| 34 | HNRNPH3 | heterogeneous nuclear ribonucleoprotein H3 [Source:HGNC Symbol;Acc:HGNC:5043] | 5745 | 0.012 | -0.2712 | No |
| 35 | PQBP1 | polyglutamine binding protein 1 [Source:HGNC Symbol;Acc:HGNC:9330] | 5827 | 0.007 | -0.2774 | No |
| 36 | RBM42 | RNA binding motif protein 42 [Source:HGNC Symbol;Acc:HGNC:28117] | 6059 | -0.009 | -0.2953 | No |
| 37 | RBFOX1 | RNA binding fox-1 homolog 1 [Source:HGNC Symbol;Acc:HGNC:18222] | 6152 | -0.016 | -0.3022 | No |
| 38 | CLK3 | CDC like kinase 3 [Source:HGNC Symbol;Acc:HGNC:2071] | 6417 | -0.037 | -0.3221 | No |
| 39 | WTAP | WT1 associated protein [Source:HGNC Symbol;Acc:HGNC:16846] | 6598 | -0.049 | -0.3352 | No |
| 40 | RBM10 | RNA binding motif protein 10 [Source:HGNC Symbol;Acc:HGNC:9896] | 6647 | -0.053 | -0.3379 | No |
| 41 | SF3B4 | splicing factor 3b subunit 4 [Source:HGNC Symbol;Acc:HGNC:10771] | 6777 | -0.061 | -0.3467 | No |
| 42 | PRDX6 | peroxiredoxin 6 [Source:HGNC Symbol;Acc:HGNC:16753] | 6783 | -0.061 | -0.3459 | No |
| 43 | RBM15B | RNA binding motif protein 15B [Source:HGNC Symbol;Acc:HGNC:24303] | 6867 | -0.069 | -0.3509 | No |
| 44 | HNRNPH2 | heterogeneous nuclear ribonucleoprotein H2 [Source:HGNC Symbol;Acc:HGNC:5042] | 7007 | -0.080 | -0.3602 | No |
| 45 | POLR2A | RNA polymerase II subunit A [Source:HGNC Symbol;Acc:HGNC:9187] | 7103 | -0.088 | -0.3658 | No |
| 46 | SMU1 | SMU1 DNA replication regulator and spliceosomal factor [Source:HGNC Symbol;Acc:HGNC:18247] | 7299 | -0.102 | -0.3790 | No |
| 47 | NOVA1 | NOVA alternative splicing regulator 1 [Source:HGNC Symbol;Acc:HGNC:7886] | 7394 | -0.110 | -0.3841 | No |
| 48 | DYRK1A | dual specificity tyrosine phosphorylation regulated kinase 1A [Source:HGNC Symbol;Acc:HGNC:3091] | 7667 | -0.132 | -0.4027 | No |
| 49 | MYOD1 | myogenic differentiation 1 [Source:HGNC Symbol;Acc:HGNC:7611] | 7839 | -0.143 | -0.4131 | No |
| 50 | HSPA1A | heat shock protein family A (Hsp70) member 1A [Source:HGNC Symbol;Acc:HGNC:5232] | 7876 | -0.146 | -0.4130 | No |
| 51 | FMR1 | FMRP translational regulator 1 [Source:HGNC Symbol;Acc:HGNC:3775] | 7944 | -0.151 | -0.4151 | No |
| 52 | RBM12B | RNA binding motif protein 12B [Source:HGNC Symbol;Acc:HGNC:32310] | 8036 | -0.158 | -0.4190 | No |
| 53 | RNPS1 | RNA binding protein with serine rich domain 1 [Source:HGNC Symbol;Acc:HGNC:10080] | 8275 | -0.177 | -0.4340 | No |
| 54 | SETX | senataxin [Source:HGNC Symbol;Acc:HGNC:445] | 8398 | -0.185 | -0.4397 | No |
| 55 | HNRNPA2B1 | heterogeneous nuclear ribonucleoprotein A2/B1 [Source:HGNC Symbol;Acc:HGNC:5033] | 8576 | -0.200 | -0.4495 | No |
| 56 | RBM8A | RNA binding motif protein 8A [Source:HGNC Symbol;Acc:HGNC:9905] | 8662 | -0.205 | -0.4520 | No |
| 57 | CLK4 | CDC like kinase 4 [Source:HGNC Symbol;Acc:HGNC:13659] | 8760 | -0.213 | -0.4552 | No |
| 58 | RBM3 | RNA binding motif protein 3 [Source:HGNC Symbol;Acc:HGNC:9900] | 8859 | -0.222 | -0.4583 | No |
| 59 | AFF2 | AF4/FMR2 family member 2 [Source:HGNC Symbol;Acc:HGNC:3776] | 8869 | -0.222 | -0.4545 | No |
| 60 | HNRNPL | heterogeneous nuclear ribonucleoprotein L [Source:HGNC Symbol;Acc:HGNC:5045] | 8892 | -0.224 | -0.4516 | No |
| 61 | RRP1B | ribosomal RNA processing 1B [Source:HGNC Symbol;Acc:HGNC:23818] | 8909 | -0.225 | -0.4482 | No |
| 62 | RBMX | RNA binding motif protein X-linked [Source:HGNC Symbol;Acc:HGNC:9910] | 8938 | -0.227 | -0.4458 | No |
| 63 | RBMY1A1 | RNA binding motif protein Y-linked family 1 member A1 [Source:HGNC Symbol;Acc:HGNC:9912] | 9014 | -0.233 | -0.4469 | No |
| 64 | HSPA8 | heat shock protein family A (Hsp70) member 8 [Source:HGNC Symbol;Acc:HGNC:5241] | 9140 | -0.243 | -0.4517 | No |
| 65 | NCBP1 | nuclear cap binding protein subunit 1 [Source:HGNC Symbol;Acc:HGNC:7658] | 9149 | -0.244 | -0.4473 | No |
| 66 | ZPR1 | ZPR1 zinc finger [Source:HGNC Symbol;Acc:HGNC:13051] | 9151 | -0.244 | -0.4424 | No |
| 67 | CDK12 | cyclin dependent kinase 12 [Source:HGNC Symbol;Acc:HGNC:24224] | 9225 | -0.250 | -0.4429 | No |
| 68 | TRA2B | transformer 2 beta homolog [Source:HGNC Symbol;Acc:HGNC:10781] | 9243 | -0.251 | -0.4391 | No |
| 69 | RBM12 | RNA binding motif protein 12 [Source:HGNC Symbol;Acc:HGNC:9898] | 9841 | -0.305 | -0.4796 | No |
| 70 | HNRNPU | heterogeneous nuclear ribonucleoprotein U [Source:HGNC Symbol;Acc:HGNC:5048] | 9847 | -0.306 | -0.4737 | No |
| 71 | PTBP3 | polypyrimidine tract binding protein 3 [Source:HGNC Symbol;Acc:HGNC:10253] | 10259 | -0.347 | -0.4988 | Yes |
| 72 | SRSF1 | serine and arginine rich splicing factor 1 [Source:HGNC Symbol;Acc:HGNC:10780] | 10272 | -0.348 | -0.4926 | Yes |
| 73 | SRSF3 | serine and arginine rich splicing factor 3 [Source:HGNC Symbol;Acc:HGNC:10785] | 10280 | -0.349 | -0.4860 | Yes |
| 74 | SRSF5 | serine and arginine rich splicing factor 5 [Source:HGNC Symbol;Acc:HGNC:10787] | 10282 | -0.349 | -0.4789 | Yes |
| 75 | RBM22 | RNA binding motif protein 22 [Source:HGNC Symbol;Acc:HGNC:25503] | 10345 | -0.355 | -0.4765 | Yes |
| 76 | HABP4 | hyaluronan binding protein 4 [Source:HGNC Symbol;Acc:HGNC:17062] | 10485 | -0.371 | -0.4797 | Yes |
| 77 | RBM15 | RNA binding motif protein 15 [Source:HGNC Symbol;Acc:HGNC:14959] | 10499 | -0.373 | -0.4731 | Yes |
| 78 | SRSF2 | serine and arginine rich splicing factor 2 [Source:HGNC Symbol;Acc:HGNC:10783] | 10507 | -0.374 | -0.4660 | Yes |
| 79 | SRPK2 | SRSF protein kinase 2 [Source:HGNC Symbol;Acc:HGNC:11306] | 10522 | -0.376 | -0.4594 | Yes |
| 80 | MBNL1 | muscleblind like splicing regulator 1 [Source:HGNC Symbol;Acc:HGNC:6923] | 10557 | -0.379 | -0.4542 | Yes |
| 81 | SRSF6 | serine and arginine rich splicing factor 6 [Source:HGNC Symbol;Acc:HGNC:10788] | 10659 | -0.389 | -0.4542 | Yes |
| 82 | YTHDC1 | YTH domain containing 1 [Source:HGNC Symbol;Acc:HGNC:30626] | 10687 | -0.391 | -0.4483 | Yes |
| 83 | RBM25 | RNA binding motif protein 25 [Source:HGNC Symbol;Acc:HGNC:23244] | 10800 | -0.405 | -0.4487 | Yes |
| 84 | CELF2 | CUGBP Elav-like family member 2 [Source:HGNC Symbol;Acc:HGNC:2550] | 10832 | -0.409 | -0.4428 | Yes |
| 85 | RBM5 | RNA binding motif protein 5 [Source:HGNC Symbol;Acc:HGNC:9902] | 10849 | -0.410 | -0.4356 | Yes |
| 86 | TIA1 | TIA1 cytotoxic granule associated RNA binding protein [Source:HGNC Symbol;Acc:HGNC:11802] | 10855 | -0.411 | -0.4276 | Yes |
| 87 | NUP98 | nucleoporin 98 and 96 precursor [Source:HGNC Symbol;Acc:HGNC:8068] | 10875 | -0.413 | -0.4206 | Yes |
| 88 | AHNAK | AHNAK nucleoprotein [Source:HGNC Symbol;Acc:HGNC:347] | 10903 | -0.417 | -0.4141 | Yes |
| 89 | SRSF10 | serine and arginine rich splicing factor 10 [Source:HGNC Symbol;Acc:HGNC:16713] | 10926 | -0.422 | -0.4072 | Yes |
| 90 | SRSF7 | serine and arginine rich splicing factor 7 [Source:HGNC Symbol;Acc:HGNC:10789] | 10930 | -0.422 | -0.3988 | Yes |
| 91 | PIK3R1 | phosphoinositide-3-kinase regulatory subunit 1 [Source:HGNC Symbol;Acc:HGNC:8979] | 11099 | -0.443 | -0.4028 | Yes |
| 92 | PRPF19 | pre-mRNA processing factor 19 [Source:HGNC Symbol;Acc:HGNC:17896] | 11269 | -0.467 | -0.4065 | Yes |
| 93 | SRRM1 | serine and arginine repetitive matrix 1 [Source:HGNC Symbol;Acc:HGNC:16638] | 11302 | -0.471 | -0.3993 | Yes |
| 94 | HNRNPF | heterogeneous nuclear ribonucleoprotein F [Source:HGNC Symbol;Acc:HGNC:5039] | 11339 | -0.476 | -0.3924 | Yes |
| 95 | METTL16 | "methyltransferase 16, N6-methyladenosine [Source:HGNC Symbol;Acc:HGNC:28484]" | 11346 | -0.478 | -0.3830 | Yes |
| 96 | CLK2 | CDC like kinase 2 [Source:HGNC Symbol;Acc:HGNC:2069] | 11368 | -0.482 | -0.3748 | Yes |
| 97 | RBM39 | RNA binding motif protein 39 [Source:HGNC Symbol;Acc:HGNC:15923] | 11434 | -0.493 | -0.3698 | Yes |
| 98 | PRX | periaxin [Source:HGNC Symbol;Acc:HGNC:13797] | 11438 | -0.494 | -0.3599 | Yes |
| 99 | CELF3 | CUGBP Elav-like family member 3 [Source:HGNC Symbol;Acc:HGNC:11967] | 11510 | -0.502 | -0.3552 | Yes |
| 100 | ZBTB7A | zinc finger and BTB domain containing 7A [Source:HGNC Symbol;Acc:HGNC:18078] | 11600 | -0.516 | -0.3515 | Yes |
| 101 | SF1 | splicing factor 1 [Source:HGNC Symbol;Acc:HGNC:12950] | 11664 | -0.527 | -0.3457 | Yes |
| 102 | DDX17 | DEAD-box helicase 17 [Source:HGNC Symbol;Acc:HGNC:2740] | 11716 | -0.535 | -0.3387 | Yes |
| 103 | SLC38A2 | solute carrier family 38 member 2 [Source:HGNC Symbol;Acc:HGNC:13448] | 11722 | -0.536 | -0.3281 | Yes |
| 104 | SRPK1 | SRSF protein kinase 1 [Source:HGNC Symbol;Acc:HGNC:11305] | 11889 | -0.569 | -0.3294 | Yes |
| 105 | METTL4 | "methyltransferase 4, N6-adenosine [Source:HGNC Symbol;Acc:HGNC:24726]" | 11976 | -0.589 | -0.3240 | Yes |
| 106 | NOVA2 | NOVA alternative splicing regulator 2 [Source:HGNC Symbol;Acc:HGNC:7887] | 12006 | -0.594 | -0.3141 | Yes |
| 107 | RBM38 | RNA binding motif protein 38 [Source:HGNC Symbol;Acc:HGNC:15818] | 12026 | -0.599 | -0.3033 | Yes |
| 108 | SNRNP70 | small nuclear ribonucleoprotein U1 subunit 70 [Source:HGNC Symbol;Acc:HGNC:11150] | 12073 | -0.613 | -0.2944 | Yes |
| 109 | PTBP2 | polypyrimidine tract binding protein 2 [Source:HGNC Symbol;Acc:HGNC:17662] | 12211 | -0.649 | -0.2918 | Yes |
| 110 | FXR2 | FMR1 autosomal homolog 2 [Source:HGNC Symbol;Acc:HGNC:4024] | 12219 | -0.650 | -0.2790 | Yes |
| 111 | U2AF2 | U2 small nuclear RNA auxiliary factor 2 [Source:HGNC Symbol;Acc:HGNC:23156] | 12226 | -0.652 | -0.2661 | Yes |
| 112 | NCL | nucleolin [Source:HGNC Symbol;Acc:HGNC:7667] | 12255 | -0.662 | -0.2547 | Yes |
| 113 | AKAP17A | A-kinase anchoring protein 17A [Source:HGNC Symbol;Acc:HGNC:18783] | 12327 | -0.684 | -0.2462 | Yes |
| 114 | RPS13 | ribosomal protein S13 [Source:HGNC Symbol;Acc:HGNC:10386] | 12334 | -0.687 | -0.2326 | Yes |
| 115 | QKI | "QKI, KH domain containing RNA binding [Source:HGNC Symbol;Acc:HGNC:21100]" | 12340 | -0.688 | -0.2188 | Yes |
| 116 | HNRNPK | heterogeneous nuclear ribonucleoprotein K [Source:HGNC Symbol;Acc:HGNC:5044] | 12341 | -0.688 | -0.2047 | Yes |
| 117 | CLK1 | CDC like kinase 1 [Source:HGNC Symbol;Acc:HGNC:2068] | 12354 | -0.693 | -0.1915 | Yes |
| 118 | HNRNPA1 | heterogeneous nuclear ribonucleoprotein A1 [Source:HGNC Symbol;Acc:HGNC:5031] | 12356 | -0.693 | -0.1773 | Yes |
| 119 | REST | RE1 silencing transcription factor [Source:HGNC Symbol;Acc:HGNC:9966] | 12382 | -0.703 | -0.1649 | Yes |
| 120 | CELF1 | CUGBP Elav-like family member 1 [Source:HGNC Symbol;Acc:HGNC:2549] | 12391 | -0.704 | -0.1510 | Yes |
| 121 | CCNL2 | cyclin L2 [Source:HGNC Symbol;Acc:HGNC:20570] | 12425 | -0.716 | -0.1389 | Yes |
| 122 | THRAP3 | thyroid hormone receptor associated protein 3 [Source:HGNC Symbol;Acc:HGNC:22964] | 12426 | -0.717 | -0.1242 | Yes |
| 123 | TRA2A | transformer 2 alpha homolog [Source:HGNC Symbol;Acc:HGNC:16645] | 12479 | -0.740 | -0.1131 | Yes |
| 124 | HNRNPH1 | heterogeneous nuclear ribonucleoprotein H1 [Source:HGNC Symbol;Acc:HGNC:5041] | 12554 | -0.776 | -0.1030 | Yes |
| 125 | JMJD6 | "jumonji domain containing 6, arginine demethylase and lysine hydroxylase [Source:HGNC Symbol;Acc:HGNC:19355]" | 12658 | -0.832 | -0.0940 | Yes |
| 126 | DAZAP1 | DAZ associated protein 1 [Source:HGNC Symbol;Acc:HGNC:2683] | 12686 | -0.854 | -0.0786 | Yes |
| 127 | CCNL1 | cyclin L1 [Source:HGNC Symbol;Acc:HGNC:20569] | 12708 | -0.870 | -0.0624 | Yes |
| 128 | FUS | FUS RNA binding protein [Source:HGNC Symbol;Acc:HGNC:4010] | 12739 | -0.902 | -0.0462 | Yes |
| 129 | SFSWAP | splicing factor SWAP [Source:HGNC Symbol;Acc:HGNC:10790] | 12747 | -0.908 | -0.0282 | Yes |
| 130 | SRSF4 | serine and arginine rich splicing factor 4 [Source:HGNC Symbol;Acc:HGNC:10786] | 12749 | -0.911 | -0.0095 | Yes |
| 131 | DDX5 | DEAD-box helicase 5 [Source:HGNC Symbol;Acc:HGNC:2746] | 12834 | -1.038 | 0.0052 | Yes |
Table: GSEA details [plain text format]

  

Fig 2: GOBP\_REGULATION\_OF\_RNA\_SPLICING      
 Blue-Pink O' Gram in the Space of the Analyzed GeneSet

  

Fig 3: GOBP\_REGULATION\_OF\_RNA\_SPLICING: Random ES distribution      
 Gene set null distribution of ES for **GOBP\_REGULATION\_OF\_RNA\_SPLICING**
